# Can GPT-5 Support Licensing Examination Preparation? Analysis of Accuracy, Reasoning, and Semantic Similarity Across Rehabilitation Disciplines

**DOI:** 10.2196/91019

**Published:** 2026-05-21

**Authors:** Christy Muasher-Kerwin, M Courtney Hughes, Aida Sanatizadeh

**Affiliations:** 1 Physical Therapy Department, North Central College, 160 E Chicago Ave, Naperville, IL, 60540, United States, 1 6306375865; 2 College of Health and Human Sciences, Northern Illinois University, Dekalb, IL, United States; 3 College of Business, Northern Illinois University, Dekalb, IL, United States

**Keywords:** large language model, clinical reasoning, licensing examination, rehabilitation education, GPT-5, semantic similarity, health professions training, artificial intelligence, board-preparation materials

## Abstract

In this cross-sectional study of 300 board-style questions across physical therapy, occupational therapy, and speech-language pathology, we evaluated reasoning types and found high overall accuracy with variation by discipline and reasoning category; the strongest performance was in deductive and analytical reasoning and the lowest accuracy was in evaluative reasoning.

## Introduction

Large language models (LLMs) such as ChatGPT (GPT-5; OpenAI) are increasingly used by clinicians and trainees to support learning and licensing examination preparation [1]. Passing licensing examinations ensures practitioner competence and patient safety and is correlated with better patient outcomes [2,3]. However, LLMs’ reasoning consistency and conceptual similarity to expert sources remain uncertain [1]. Some prior research has demonstrated that LLMs can achieve passing scores on various professional examinations, but their explanations may exhibit shallow logic or inconsistent reasoning [4]. Recent evaluations of ChatGPT on Chinese national licensing examinations similarly demonstrated failure to meet official pass thresholds, with accuracy varying across examination type and question format [5]. Our study assessed GPT-5’s accuracy, reasoning patterns, and semantic similarity to verified board-preparation materials for rehabilitation examinations for physical therapy (PT), occupational therapy (OT), and speech-language pathology (SLP) to assess whether GPT-5 can serve as a reliable study assistant.

## Methods

### Overview

Three hundred multiple-choice questions (100 each from PT, OT, and SLP validated board-preparation sources that are not publicly available) were entered into GPT-5 (via the ChatGPT web interface) in November 2025 using default settings, without hints or chain-of-thought prompting, to mimic typical learner use. For each discipline, the first 100 sequential questions were included to create a reproducible sample. Difficulty indices and blueprint mappings were unavailable; therefore, items were not stratified.

Selected answers from the model and written rationales were compared with source materials. Accuracy was calculated across items and recorded as correct or incorrect. Rationales were coded by reasoning type: inductive (from specific examples to a general conclusion), deductive (from a general rule to a specific conclusion), analytical (examining parts and their relationships), evaluative (judging something based on criteria), or inferential (drawing a conclusion from evidence). This coding was based on the cognitive process required for the correct answer. Reasoning classifications were predefined within the board-preparation materials and aligned with established educational frameworks.

Semantic similarity was computed using cosine similarity applied to L2-normalized sentence embeddings generated by the all-MiniLM-L6-v2 Sentence-BERT model, and preprocessing was limited to minimal white space normalization, consistent with recommended Sentence-BERT use [6]. Descriptive statistics summarized performance across disciplines. Incorrect responses were qualitatively reviewed to identify recurring conceptual challenges. A single coder (CM-K) conducted inductive content analysis using an emergent coding approach [7] and grouped content by shared subject matter to identify the specific content domains that presented greater difficulty for GPT-5. Initial codes were developed through iterative review of incorrect responses and GPT-5 rationales using constant comparison. A second coder (MCH) reviewed categories, and disagreements were discussed and resolved. Because coding focused on thematic categorization of recurring errors, formal interrater reliability statistics were not calculated. Consensus review was used to ensure interpretive agreement and consistency of category definitions prior to final theme reporting.

### Ethical Considerations

This project used publicly available, non–human participant material and did not require institutional review board approval.

## Results

GPT-5 demonstrated strong factual accuracy overall, although performance varied by discipline and reasoning type, with only deductive reasoning in PT receiving 100% (Table 1). Overall accuracy for GPT-5 was 91.0% for PT (95% CI 83.6%-95.8%), 79.0% for OT (95% CI 68.9%-85.5%), and 83.0% for SLP (95% CI 74.4%-89.6%).

**Table 1.** Accuracy by discipline and reasoning type.

Reasoning type	Physical therapy (n=100), n/N (%)	Occupational therapy (n=100), n/N (%)	Speech-language pathology (n=100), n/N (%)
Deductive	22/22 (100/100)	11/15 (73.3/100)	31/33 (93.9/100)
Analytical	22/23 (95.7/100)	9/11 (81.8/100)	40/47 (84.8/100)
Inductive	19/22 (86.4/100)	18/21 (85.7/100)	2/4 (50.0/100)
Inferential	22/25 (88.0/100)	26/36 (72.2/100)	6/9 (66.7/100)
Evaluative	6/8 (75/100)	15/17 (88.2/100)	4/7 (57.1/100)
Overall accuracy	(91.0/100)	(79.0/100)	(83.0/100)

Across all questions, the mean semantic similarity between GPT-5 and source-material rationales was 0.707. This indicates semantic alignment between GPT-5 rationales and expert rationales. Deductive reasoning had the highest mean similarity (0.730), followed by analytical (0.714), inductive (0.675), inferential (0.685), and evaluative (0.671) reasoning. The Kruskal-Wallis *H* test across all 5 reasoning types was also significant: *H*_4_=11.7 (*P*=.02). This suggests that GPT-5’s explanations aligned more closely with reference rationales for deductive reasoning tasks, where rules are applied to reach conclusions, than for tasks requiring generalization, inference, or evaluation.

Qualitative review of incorrect answers identified recurring conceptual challenges. Using inductive content analysis, challenges were grouped by shared conceptual features in GPT-5 rationales. Categories appearing across multiple questions within a discipline are summarized in Textbox 1.

Textbox 1. **Summary of missed question-type patterns across disciplines.**

**Physical therapy**

- Functional reasoning (3/9, 33.3%)
- Safety/priority decision-making (3/9, 33.3%)
- Technical recall (3/9, 33.3%)

**Occupational therapy**

- Functional reasoning (12/17, 70.6%)
- Professional scope and delegation reasoning (3/17, 17.6%)
- Safety-based decision-making (2/17, 11.8%)

**Speech-language pathology**

- Interpretive clinical reasoning for specialized assessment and instrumental findings (10/22, 45.5%)
- Evidence-based intervention selection (7/22, 31.8%)
- Procedural and ethical decision-making (5/22, 22.7%)

## Discussion

In this evaluation of board-preparation materials, GPT-5 reproduced knowledge required for entry-level competence but continued to show deficits in higher-order reasoning. Because reasoning categories reflect examination item demands rather than GPT-5’s internal reasoning, findings represent performance patterns across question types. Although semantic similarity to expert rationales was high, reasoning lapses suggest that the model captures conceptual language patterns without consistently modeling clinicians’ inferential logic. These findings reinforce that semantic similarity reflects linguistic alignment rather than equivalence in clinical reasoning. These findings extend previous work showing that LLMs can approach human-level factual performance while still lacking in cognitive depth and explainability [8].

Findings should be interpreted cautiously because board-preparation materials differ from official licensing examinations, which undergo formal psychometric validation, calibrated difficulty scaling, and secure item rotation. Thus, results do not represent performance on live examinations. Although accuracy approached commonly cited passing thresholds [9], comparisons are limited by scoring differences across professions.

Given the shift toward competency-based medical education and assessment frameworks, such as entrustable professional activities (EPAs) [10], the reasoning deficits observed here raise important questions about whether LLMs can support learners’ progression toward entrustment. In structured settings, GPT-5 may be used as a formative adjunct, with educators leveraging model rationales to stimulate reflection and compare reasoning against established milestones or EPAs. Such supervised use supports competency development with oversight. While GPT-5 reproduced factual knowledge, its lapses in inferential and evaluative reasoning highlight limitations in supporting competencies related to clinical judgment, prioritization, and ethical decision-making.

This study is limited by its sample size, focus on rehabilitation disciplines only, and absence of learner outcome data. Because reasoning patterns may differ across specialties, future research should evaluate larger question sets, additional medical domains, intermodel comparisons, and performance across LLM models and prompting conditions. Evaluating LLM reasoning in interactive or case-based formats may reflect clinical decision tasks. Finally, longitudinal studies should examine how learners use LLMs during examination preparation to determine whether model reasoning errors influence learner understanding or create opportunities for instructional interventions.

Although GPT-5 reproduced substantial factual content, observed reasoning gaps support supervised integration in examination preparation. Because this study evaluated examination performance rather than clinical outcomes, any safety implications are theoretical. AI tools should complement, not replace, educator-guided reasoning development.

## Abbreviations

EPA: entrustable professional activity
LLM: large language model
OT: occupational therapy
PT: physical therapy
SLP: speech-language pathology
